# Chronic pain as a brain imbalance between pain input and pain suppression

**DOI:** 10.1093/braincomms/fcab014

**Published:** 2021-02-16

**Authors:** Sven Vanneste, Dirk De Ridder

**Affiliations:** 1 Global Brain Health Institute, Institute of Neuroscience, Trinity College Dublin, Dublin, Ireland; 2 Department of Surgical Sciences, Section of Neurosurgery, Dunedin School of Medicine, University of Otago, 9016 Dunedin, New Zealand

**Keywords:** pain, medial pathway, lateral pathway, descending pathway, anterior cingulate cortex

## Abstract

Chronic pain is pain that persists beyond the expected period of healing. The subjective experience of chronic pain results from pathological brain network interactions, rather than from persisting physiological sensory input of nociceptors. We hypothesize that pain is an imbalance between pain evoking dorsal anterior cingulate cortex and somatosensory cortex and pain suppression (i.e. pregenual anterior cingulate cortex). This imbalance can be measured objectively by current density ratios between pain input and pain inhibition. A balance between areas involved in pain input and pain suppression requires communication, which can be objectively identified by connectivity measures, both functional and effective connectivity. In patients with chronic neuropathic pain, electroencephalography is performed with source localization demonstrating that pain is reflected by an abnormal ratio between the dorsal anterior cingulate cortex, somatosensory cortex and pregenual anterior cingulate cortex. Functional connectivity demonstrates decreased communication between these areas, and effective connectivity puts the culprit at the dorsal anterior cingulate cortex, suggesting that the problem is related to abnormal behavioral relevance attached to the pain. In conclusion, chronic pain can be considered as an imbalance between pain input and pain suppression.

## Graphical Abstract

**Graphical Abstract:**
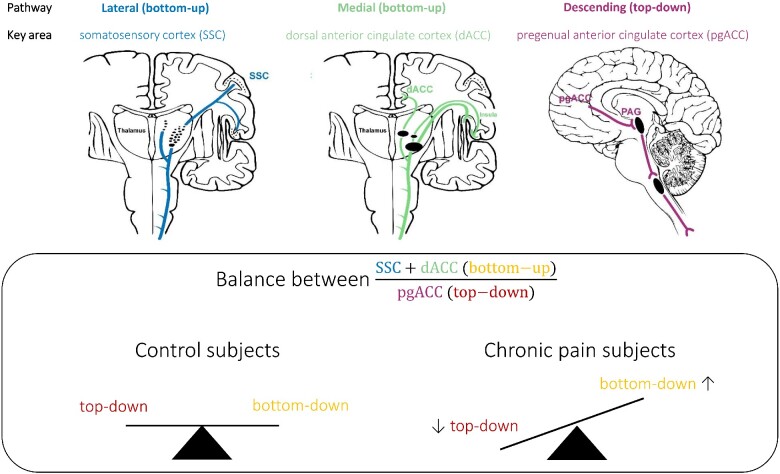

**Abbreviated summary**

Vanneste & De Ridder introduce a new concept of chronic pain as a brain imbalance disorder, demonstrating that pain is reflected by an abnormal ratio between frontal brain areas, that goes together with decreased communication between these areas, suggesting the dorsal anterior cingulate cortex as the culprit.

## Introduction

Over one-third of the world's population suffers from persistent or chronic pain, resulting in tremendous burden for the individual (Jensen *et al.*, 2014) and society (Gaskin and Richard, 2012). The subjective experience of chronic pain results from pathological brain network interactions, rather than from persisting physiological sensory input via nociceptors (Jensen *et al.*, 2014). Pain is processed by three known pathways (Rainville *et al.*, 1997; Price, 2000; Fields, 2004; Eippert *et al.*, 2009; Kong *et al.*, 2010; De Ridder and Vanneste, 2016). The two ascending pathways include the anatomically and functionally separable medial and lateral pain pathway (Price, 2000; Bushnell *et al.*, 2013). The medial pain pathway, which involves the dorsal anterior cingulate cortex (dACC) and anterior insula as main hubs, encodes the unpleasantness and suffering of pain (Rainville *et al.*, 1997; Price, 2000; Bushnell *et al.*, 2013). The lateral pathway, which involves the somatosensory cortex (SSC) as major hub, processes the discriminatory/sensory components of the pain (Flor *et al.*, 1995; Bushnell *et al.*, 2013). In addition, a descending pain inhibitory pathway (Fields, 2004) involves the rostral and pregenual anterior cingulate cortex (pgACC), the periaqueductal gray, hypothalamus and rostral ventromedial brainstem (Fields 2004; Eippert *et al.*, 2009; Kong *et al.*, 2010). This descending pathway is responsible for stress-mediated pain inhibition (Yilmaz *et al.*, 2010), and placebo analgesia (Eippert *et al.*, 2009).

It has been proposed that pain is the result of an imbalance between brain areas that process pain input and pain suppression (De Ridder and Vanneste, 2016), and that if pain input equals pain suppression there is no pain perception, but if pain input is larger than pain suppression this results in subjective pain perception (De Ridder and Vanneste, 2016). This imbalance can be computed by analyzing the current density of the different frequency bands of the electroencephalogram (EEG) spectrum, source localized to the pgACC, dACC and SSC (De Ridder and Vanneste, 2016).

The frequency bands of interest within the EEG spectrum are θ, α and γ band activity, based on the thalamocortical dysrhythmia model of pain (Llinas *et al.*, 1999; Sarnthein *et al.*, 2005; Stern *et al.*, 2006; Cauda *et al.*, 2009; Walton *et al.*, 2010; De Ridder *et al.*, 2011; Schulman *et al.*, 2011; Sametsky *et al.*, 2015; Vanneste *et al.*, 2018). The thalamocortical dysrhythmia hypothesis suggests that somatosensory deafferentation leads to a thalamocortical column-specific decrease in information processing, which permits slowing down of resting-state thalamocortical activity from normal α to the θ frequency range (Llinas *et al.*, 1999, 2005; Vanneste *et al.*, 2018). Decreased input also results in a reduction of GABA_A_-mediated lateral inhibition, inducing γ band activity surrounding the deafferented thalamocortical columns (Llinas *et al.*, 2005). This γ band activity surrounding θ activity is known as the edge effect (Llinas *et al.*, 1999, 2005). An abnormal increase of θ oscillations in chronic pain patients has been identified (Sarnthein *et al.*, 2006; Stern *et al.*, 2006). Other studies, however, did not observe abnormal θ oscillations in chronic pain (Schmidt *et al.*, 2012; Jensen *et al.*, 2013), but only a slowing of the peak α frequency in chronic pain (de Vries *et al.*, 2013). Furthermore, abnormal θ oscillations might basically represent the non-specific slowing of α activity observed in many chronic neurological and psychiatric disorders (Vanneste *et al.*, 2018).

It has been proposed that the θ-γ coupling in thalamocortical dysrhythmia represents pain-related γ activity (Babiloni *et al.*, 2002) nested on θ as a carrier wave (Lisman and Jensen, 2013), analogous to what has been proposed for cognitive processing (Canolty *et al.*, 2006). This γ activity nested on θ is also known in other pathologies such as tinnitus, depression, Parkinson’s disease, addiction, ADHD, etc. (Coullaut-Valera *et al.*, 2014; De Ridder *et al.*, 2015; Kim *et al.*, 2015, 2017; Mumtaz *et al.*, 2017; Vanneste *et al.*, 2018).

A balance between pain input and pain suppression requires communication between the involved areas. Communication in the brain can be analyzed by functional connectivity, effective connectivity and cross-frequency coupling. Functional connectivity reflects correlated activity between two or more areas in the brain, effective connectivity adds directionality to the functional connectivity and cross-frequency coupling looks at how high frequency activity (β and γ) is nested on low frequency activity (δ, θ, α).

This study wants to confirm or disprove the proposed brain imbalance model for pain using EEG to analyze source localized activity (=current density) and connectivity. Based on the thalamocortical dysrhythmia model for pain the frequencies of interest are θ, α and γ, the regions of interest are the main hubs of the lateral pathway (=SSC), the medial pathway (=dACC) and descending pain inhibitory pathway (=pgACC). We hypothesize that pain is associated with an increased ratio >1 of the current density of (dACC+SSC)/2×pgACC, that the functional and effective connectivity is disrupted in the θ and α band, and the cross-frequency coupling abnormal in θ-γ.

## Methods and materials

### Participants

This study consists of 100 subjects (50 women and 50 men; 34–66 years of age, *M *=* *48.47; *SD* = 11.04): 50 healthy control subjects, and 50 subjects with chronic pain. The healthy control group reported no history of neurological or neuropsychiatric disorders, and no pain. A pain specialist screened the pain patients for neuropathic pain related to deafferentation (i.e. peripheral nerve, root, or central tract lesions). Whereas traditionally chronic pain is defined as pain lasting for more than 3 months (Treede *et al.*, 2015), the patients in this study suffered pain for more than one year.

The pain score (pain percept: how severe is your pain?) on a numeric rating scale was 5.80 (*SD* = 2.57) and on the pain vigilance and awareness questionnaire (PVAQ) 47.01 (*SD* = 9.41). The PVAQ is a measurement that assesses the preoccupation with or attention to pain and pain changes, and is associated with pain-related fear and perceived pain severity (Roelofs *et al.*, 2003). The study was in accordance with the ethical standards of the Helsinki declaration (1964) and was approved by the institutional ethics committee (UZA OGA85). All relevant data are available from the authors on request.

### Electroencephalogram

#### Recordings

EEG data (Neuroscan, http://compumedicsneuroscan.com/) were obtained in a quiet room while each participant was sitting upright on a comfortable chair. The EEG was recorded with 64 electrodes in the standard 10–10 International placement. The impedances were checked to remain below 5 kΩ. Data recording was eyes-closed (sampling rate = 1 kHz, band passed DC–200 Hz) and lasted approximately 5 min. The ground electrode was located at AFZ and the reference was located at the vertex. Participants were instructed not to drink caffeinated beverages one hour and alcohol 24 hours prior before recording to avoid alcohol- or caffeine-induced changes in the EEG stream (Volkow *et al.*, 2000; Logan *et al.*, 2002; Siepmann and Kirch, 2002). By monitoring both slowing of the alpha rhythm and appearance of spindles in the EEG stream we checked the alertness of participants to prevent possible enhancement of the θ power due to drowsiness during recording (Moazami-Goudarzi *et al.*, 2010). Our data were resampled to 128 Hz, band-pass filtered in the range 2–44 Hz, plotted and carefully inspected for manual artifact-rejection. We removed all episodic artifacts including eye blinks, eye movements, teeth clenching, body movement, or ECG artifact from the stream of the EEG. We computed the average Fourier cross-spectral matrices for frequency bands δ (2–3.5 Hz), θ (4–7.5 Hz), α (8–12 Hz), β (13–30 Hz) and γ (30.5–44 Hz).

#### Source localization analysis

Standardized low-resolution brain electromagnetic tomography (sLORETA, available at http://www.uzh.ch/keyinst/loreta.htm) is a method for functional imaging yielding standardized current density with zero localization error based on certain electrophysiological and neuroanatomical constraints (Pascual-Marqui, 2002). This method is developed to estimate the intracerebral sources generating the scalp-recorded electrical activity in the five frequency bands defined (Song *et al.*, 2015). The sLORETA algorithm solves the inverse problem by assuming related orientations and strengths of neighboring neuronal sources that are represented by adjacent voxels. The solution space used in this study and associated lead field matrix are those implemented in the LORETA-Key software*.* By applying the boundary element method on the MNI-152 (Montreal Neurological Institute, Canada) sLORETa implements revisited realistic electrode coordinates (Jurcak *et al.*, 2007) and the lead field (Fuchs *et al.*, 2002) on a MNI-152 volume with 6239 voxels together with a size of 5 × 5 × 5 mm. The co-registration makes use of the correct translation from the MNI-152 space into the Talairach and Tournoux space.

#### Region of interest analysis

The log-transformed electric current density was calculated for each regions of interest (ROIs) and averaged across all voxels belonging to the ROI for the fivet frequency bands included in this study. The ROIs in the present study are the left and right SSC, the dACC, and pgACC. We did not differentiate between left and right dACC, pgACC due to their proximity to the midline, as due to volume conduction, laterality is harder to differentiate for areas close to the midline.

#### Support vector machine

We used the SVM program in the data-mining software Weka to perform all classification tasks (Waikato Environment for Knowledge Analysis version 3.7, developed by the University of Waikato Machine Learning Group, available at http://www.cs.waikato.ac.nz/ml/weka/) (Smith and Frank, 2016) We build a predictive models by learning from examples provided in user supplied datasets and used the default settings as the running parameters. Our dataset included for each participant the five frequency bands for each ROI. The criterion for correct classification was defined by subjects being assigned to the correct group (e.g. for the full model: disorder versus healthy). The classification method that was used, was a linear logistic regression-based classifier as the classification method. A 10-fold cross-validation was performed on the full dataset (see Vanneste *et al.*, 2018 for more detail information). The measurements of model accuracy calculated by the *k*-fold cross-validation technique include the true-positive ratio (TPR), false-positive ratio (FPR), root mean squared error (RMSE), mean average error (MAE), and κ-statistic (see Vanneste *et al.*, 2018 for more detail information about this specific measures). In order to determine significance of the model accuracy for pain/control, averaged model accuracy was calculated through randomization of the data. This was done by taking the same dataset used to generate the pain model and randomly reassigning patient data as either pain or control. This randomized dataset was then used to generate a prediction model and to model accuracy values. This was done 100 times, and the resulting randomized model accuracy statistics were averaged across all trials.

#### Lagged phase coherence

Phase coherence between time series corresponding to different spatial locations are usually interpreted as indicators of functional connectivity. Pascual-Marqui (2007a,b) introduced measures of coherence and phase synchronization that takes into account only non-instantaneous connectivity. Because this method the confounding factor of volume conduction is removed. The lagged phase coherence between two sources can then be interpreted as the amount communication between the regions contributing to the ROI defined. Since the two components oscillate coherently with a lag, the crosstalk can be interpreted as information sharing (more detail information can be found in Vanneste *et al.*, 2014).

#### Granger causality

Granger causality reflects information transfer strength also called effective connectivity. By quantifying how much the signal in the seed region is able to predict the signal in the target region we calculated the effective connectivity from one region to another (Granger, 1969; Geweke, 1982). Granger causality is defined as the log-ratio between the error variance of a reduced model, which predicts one time series based only on its own past values, and that of the full model, which in addition includes the past values of another time series. In this study, we look at the granger causality between the pgACC, dACC, and the left and right SSC.

#### Cross-frequency coupling

θ-β, θ-γ and α-γ coupling are proposed to be effective means of communication between cortically distant areas (Canolty *et al.*, 2006). To verify whether this is present, cross-frequency coupling was calculated for the pgACC, dACC, and the left and right SSC. Phase–amplitude was computed by computing the time-series for the *x*, *y* and *z* components of the current for each ROI. Next, these were filtered in the θ, α, β and γ frequency band-pass regions. For each ROI and each frequency band, a principal component analysis was calculated, and the first component was retained for the θ and γ bands. A Hilbert transform was then calculated on the gamma component and the signal envelope retained. Finally, the Pearson correlation between the θ/α component and the envelope of the β/γ envelope was computed for each participant.

### Statistical analysis

#### Support vector machine

To compare the different outcome measures (correctly classified, incorrectly classified, TPR, FPR, ROC, κ-statistic, RMSE and MAE) of the SVM learning approach, we applied a univariate ANOVA with the model (test versus random) as the independent variable and the outcome measures as the dependent variable.

#### Region of interest

We performed a MANOVA including the log-transformed current density for the pgACC, dACC and the left and right SSC as dependent variables and group (chronic pain patients versus control subjects) as independent variables. These areas were selected based on the a priori hypothesis and were confirmed by SVM. For each area, we analyzed only the frequencies that showed up when applying in the SVM learning approach. Based on the outcome, a simple contrast analyses were conducted to look at specific effects for a specific frequency and a specific area. Pearson correlations were calculated between the region of interest and the pain score for the frequency bands at the areas that showed up during SVM. This analysis was corrected for pairwise comparisons using a Bonferroni correction. In addition, we looked at the balance between the areas that are part of the ascending pathway (i.e. dACC and the left/right SSC) and the descending pathway (i.e. pgACC). We include only the frequency bands that correlated with the pain percept as measured with the visual analogue scale. As the dACC and SSC combine two current densities and the pgACC only one, the current density of the pgACC was doubled, as such, the balance was calculated as follows:

$$\frac{\left(\frac{\beta |\mathrm{dACC}+(\frac{\gamma |\mathrm{left}\;\mathrm{SCC}+\gamma |\mathrm{right}\;\mathrm{SCC}}{2})}{2}\right)}{\theta |\mathrm{pgACC}}$$

If the balance was greater than one, then it suggests that the key areas involved in the ascending pathways are more active in comparison to the key area involved in the descending pathway. If the balance was less than one, then it suggests that the key areas involved in the ascending pathways are less active in comparison to the key area involved in the descending pathway. If the balance is similar to one, then the key areas involved in the ascending pathways are as active as the key area involved in the descending pathway. A univariate ANOVA was applied to compare the balance between the control subjects and the chronic pain patients. In addition, a Pearson correlation was applied between the pain percept and the balance score.

#### Lagged phase coherence or functional connectivity

A comparison was made between chronic pain patients versus control subjects for all frequency bands. The threshold of significance for a given lagged phase coherence can be found as described by Pascual-Marqui (2002, 2007a,b). Time-series of current density were extracted for different regions of interest using sLORETA. Power in all 6239 voxels was normalized to a power of 1 and log transformed at each time point. Region-of-interest values thus reﬂect the log transformed fraction of total power across all voxels, separately for specific frequencies. The regions of interest selected were the pgACC, dACC, and left and right SSC. Lagged phase synchronization/coherence or functional connectivity contrast maps were calculated for all frequency bands: δ (2–3.5 Hz), θ (4–7.5 Hz), α (8–12 Hz), β (13–30 Hz) and γ (30.5–44 Hz). The significance threshold was based on a permutation test with 5000 permutations. This methodology corrects for multiple testing (i.e. for the collection of tests performed for all voxels, and for all frequency bands).

#### Granger causality

We applied Granger causality analysis to look at the direction of functional connectivity (i.e. effective connectivity). For the θ frequency, we performed a MANOVA including the Granger causality for the effective connectivity (pgACC→left SSC, left SSC→pgACC, pgACC→right SSC, and right SSC→pgACC) as dependent variables, and group (chronic pain patients versus control subjects) as independent variables. Based on the outcome, simple contrast analyses were conducted for the individual connections. For the alpha frequency, we performed a MANOVA including the effective connectivity (pgACC→left SSC, left SSC→pgACC, pgACC→right SSC, right SSC→pgACC, pgACC→dACC, dACC→pgACC, dACC→left SSC, left SSC→dACC, dACC→right SSC, right SSC→dACC, left SSC→right SSC and right SSC→left SSC) as dependent variables and group (chronic pain patients versus control subjects) as independent variables. Based on the outcome, simple contrast analyses were conducted for the individual connections. We calculated Pearson correlations between effective connectivity and pain perception for all connections that showed a significant group-level effect.

#### Cross-frequency coupling

We performed a MANOVA including θ-γ and α-γ phase-amplitude coupling for the both the left and right SSC and α-β coupling for the dACC as dependent variables and group (chronic pain patients versus control subjects) as independent variables. Based on the outcome, simple contrast analyses were conducted to look at specific effects. A Pearson correlation was computed between θ-γ and α-γ coupling for the left and right SSC for both the chronic pain patients and control subjects. Furthermore, a Pearson correlation was calculated between the phase-amplitude coupling for θ-γ and α-γ for the SSC, the α-βcoupling for the dACC, and pain perception as well as the balance between θ-γ and α-γ for the SSC.

### Data availability

Data will be made available upon reasonable request.

## Results

### Support vector machine learning

**Figure 1:**
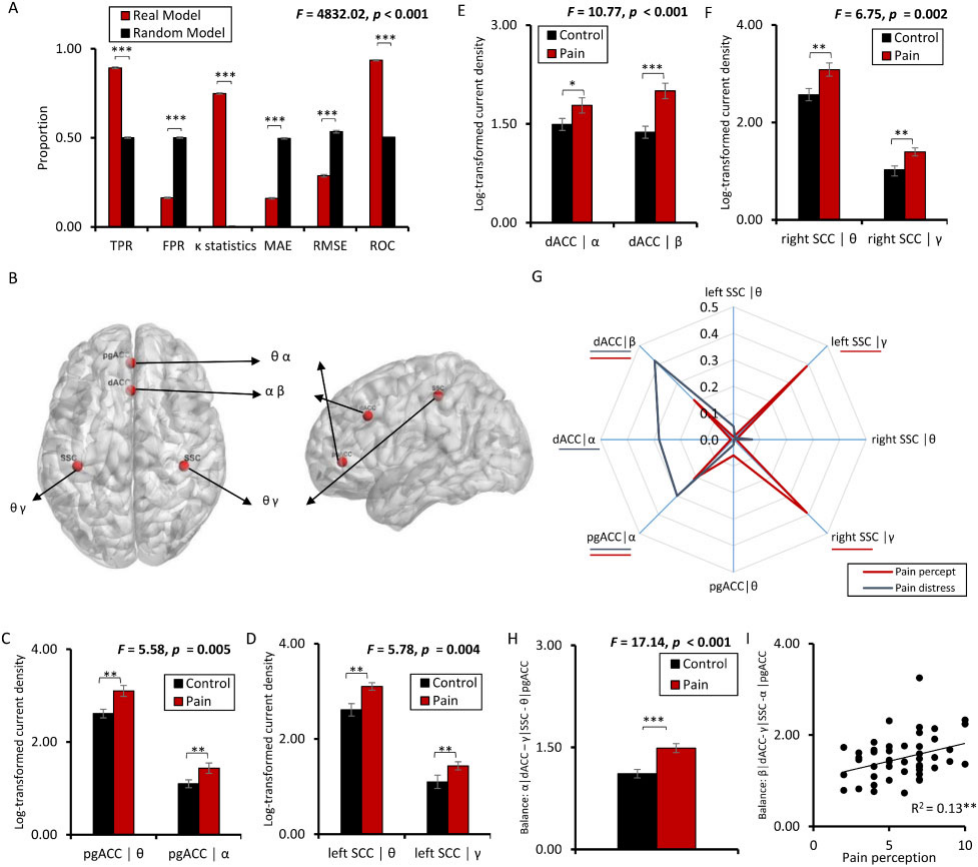
**Activity changes**. (A) Support vector machine (SVM) learning differentiating between chronic pain and healthy controls with an accuracy 89.3% in comparison to a random model. The true positive rate (TPR) and the area under the curve (ROC) were significantly higher for the obtained model in comparison to the random model, while the false positive rate (FPR) was significantly lower. A significant difference was also identified by comparing the κ-statistic, MAE and RMSE, confirming the strength of the tested model in comparison to the random model. (B) SVM differentiates between respectively chronic pain versus controls for the pregenual anterior cingulate cortex (pgACC), for the θ and α frequency band, dorsal anterior cingulate cortex (dACC) for the α and β frequency band and for the left and right somatosensory cortex (SSC) for the θ and γ frequency band. (C–F) Activity changes in the areas obtained with SVM show significant increase in chronic pain patients in comparison to control subjects for the specific frequency band obtained with SVM. (G) Correlations between the pain intensity percept (red) and the activity for the pgACC, dACC, left and right SSC and correlations between the pain distress (black) and the activity for the pgACC, dACC, left and right SSC reveals. (H) The difference in balance between the pgACC at α, and the dACC at β plus the left and right SCC at γ frequency band is significantly increased in patients with chronic pain in comparison to control subjects suggesting that the key areas (dACC and SCC) involved in the ascending pathways are more active in comparison to the key area involved in the descending pathway. (I)This imbalance correlates with the perceived pain intensity. (†*P  *<* *0.10, *P <* *0.05, **P <* *0.01, ***P <* *0.001). For the radar plots, effective connectivity areas underlined indicate a significant effect (blue for pain distress, red for pain intensity).

Using a SVM learning approach, chronic pain (*n* = 50) can be objectively detected in comparison to pain-free control subjects (*n* = 50), solely based on brain activity of the dACC (including α and β band), the SSC (including θ and γ band), and the pgACC (including θ and α band) with an accuracy of 89.3%. The other frequency bands, namely delta and beta, were not selected by the model. A random model has an accuracy not higher than chance (TPR: 50%) (*F *=* *2593.09, *P* < 0.001) (Fig. 1a, b). The FRP was significantly (*F *=* *22772.37, *P* < 0.001) lower for the pain model (*M* = 0.16, *SD* = 0.02) in comparison to the random model (*M* = 0.50%, *SD* = 0.02). The area under the curve shows a significant effect (*F *=* *7281.34, *P* < 0.001), indicating a higher score for the pain model (*M* = 0.94, *SD* = 0.003) in comparison to the random model (*M* = 0.50, *SD* = 0.003). A significant difference was also obtained by comparing the κ-statistic (pain: *M* = 0.75, *SD* = 0.02 versus random: *M* = 0.002, *SD* = 0.004; *F *=* *13404.91, *P* < 0.001), MAE (pain: *M* = 0.16, *SD* = 0.005 versus random: *M* = 0.50, *SD* = 0.005; *F *=* *2299.95, *P* < 0.001), and RMSE (pain: *M* = 0.29, *SD* = 0.007 versus random: *M* = 0.54, *SD* = 0.007; *F *=* *436.24, *P* < 0.001)) (Fig. 1a, b). For the areas individually, the maximum accuracy was 68.8% (dACC: 67.4%; the SSC: 68.8%; pgACC: 63.3%) and the accuracy for the dACC and SSC together was 79.3%, confirming that pain can better be explained by looking at the pain input and suppression areas together (89.3% accuracy), i.e. as a balance.

### Region of interest

A region of interest analysis further shows that chronic pain is the result of an increase in activity in the SSC (θ and γ band; left: *F *=* *5.78, *P* = 0.004; right: *F *=* *6.75, *P* = 0.002), dACC (α and β band; *F *=* *10.77, *P* < 0.001), and pgACC (θ and α band; *F *=* *5.58, *P* = 0.005) (Fig. 1c–f). These effects remained after correction for multiple comparison using a Bonferroni correction (*P* < 0.016). A significant increase for the pain patients in the left (θ: *M *=* *3.10, *SD* = 1.06, *F *=* *6.48, *P* = 0.010; γ: *M *=* *1.43, *SD* = 0.72, *F *=* *8.36, *P* = 0.005) and right SCC (θ: *M *=* *3.08, *SD* = 1.01, *F *=* *7.82, *P* = 0.006; γ: *M *=* *1.40, *SD* = 0.67, *F *=* *10.29, *P* = 0.002) was obtained in comparison to the control group (left SCC for θ: *M *=* *2.61, *SD* = 0.92; and for γ: *M *=* *1.010, *SD* = 0.48 and right SCC for θ: *M *=* *2.57, *SD* = 0.90; for γ: *M *=* *1.02, *SD* = 0.50), respectively. For the pgACC, both for the θ and α band a significant increase in current density was identified for pain patients (θ: *M *=* *3.10, *SD* = 0.89, *F *=* *7.64, *P* = 0.009; α: *M *=* *1.44, *SD* = 0.84, *F *=* *8.13, *P* = 0.005) in comparison to the control subjects (θ: *M *=* *2.61, *SD* = 0.65; α: *M *=* *1.10, *SD* = 0.61). Also, a comparison between the current density for the dACC for the α and β band revealed a significant increase for the pain patients (α: *M *=* *1.78, *SD* = 0.87, *F *=* *5.53, *P* = 0.039; β: *M *=* *2.01, *SD* = 0.86, *F *=* *13.25, *P* < 0.001) in comparison to the control subjects (α: *M *=* *1.49, *SD* = 0.63; β: *M *=* *1.37, *SD* = 0.64). These effects remained after correction for multiple comparison using a Bonferroni correction (*P* < 0.0125).

The current density correlates with the pain percept in γ for left SSC (γ: *R*^2^ = 0.39, *P* < 0.001) and right SSC (γ: *R*^2^ = 0.39, *P* < 0.001), in β for the dACC (β: *R*^2^ = 0.21, *P* = 0.007) and alpha for pgACC (α: *R*^2^ = 0.21, *P* = 0.007), but not for θ, neither in the left SSC (θ: *R*^2^ = 0.01, *P* = 0.46), right SSC (θ: *R*^2^ = 0.01, *P* =0.65), nor pgACC (θ: *R*^2^ = 0.06, *P* = 0.43), and neither for α in the dACC (α: *R*^2^ = 0.01, *P* = 0.08) (Fig. lg). These effects continued after correction for multiple comparison using a Bonferroni correction (*P* < 0.008).

A correlation analysis was performed between the current density and pain severity/distress as measured by the pain vigilance and awareness questionnaire. This analysis revealed a significant positive correlation between the current density in α in the pgACC (α: *R*^2^ = 0.30, *P* < 0.001) and α and β in the dACC (α: *R*^2^ = 0.28, *P* < 0.001| β: *R*^2^ = 0.42, *P* < 0.001) indicating the higher the score for pain related distress, the higher the current density, or vice versa. No effect was obtained for left SSC (θ: *R*^2^ = 0.05, *P* = 0.32| γ: *R*^2^ = 0.01, *P* = 0.37) and right SSC (θ: *R*^2^ = 0.07, *P* = 0.07|) γ: *R*^2^ = 0.001, *P* = 0.87) and the pgACC (θ: R^2^ = 0.02, *P* = 0.26) (Fig. lg). The effects continued after correction for multiple comparison using a Bonferroni correction (*P* < 0.008).

The balance between the areas of interest (pgACC, dACC and SSC) is significantly different from healthy pain-free subjects (*F *=* *17.14, *P* < 0.001) and furthermore correlates with intensity of the pain (*R*^2^ = 0.13, *P* = 0.012) (Fig. lh–i). No correlation was obtained between the balance between these significant areas and pain distress (*R*^2^ = 0.07, *P* = 0.08). After correction for multiple comparisons using a Bonferroni correction the effects remained (*P* < 0.025).

### Functional connectivity

**Figure 2:**
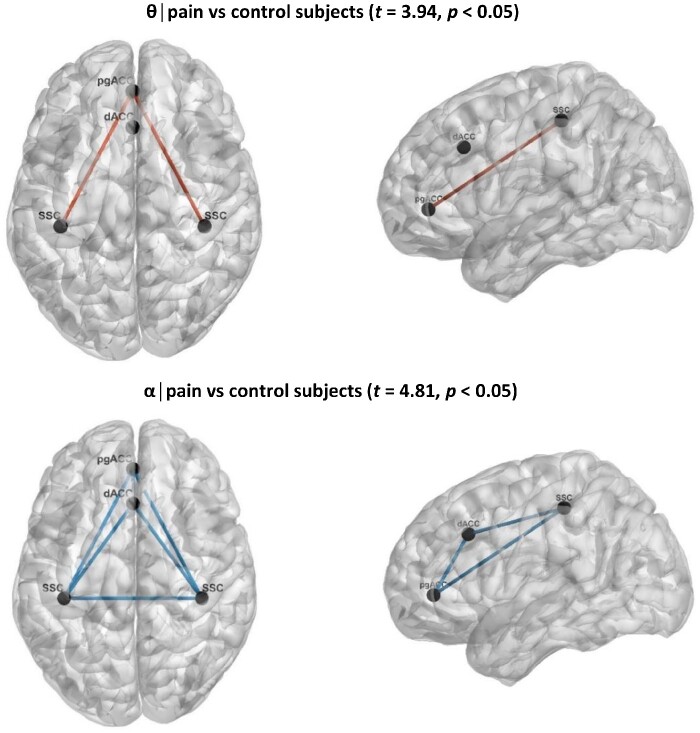
**Functional connectivity in pain**. Functional connectivity between patients with chronic pain in comparison to control subjects shows a significant change in the θ and α frequency band. For θ, a significant increase in functional connectivity was revealed between the pgACC and the left and right SSC for chronic pain patients, while for α a decreased functional connectivity was revealed between the pgACC, dACC and the left and right SSC for chronic pain patients.

Functional connectivity, based on lagged phase synchronization between the ROI brain areas demonstrates that chronic pain patients have decreased functional connectivity in the α band (*t *=* *3.94, *P* < 0.05) and more functional connectivity in the θ band (*t *=* *4.81, *P* < 0.05) (Fig. 2). No effect was obtained for δ, β and γ bands.

### Effective connectivity

**Figure 3:**
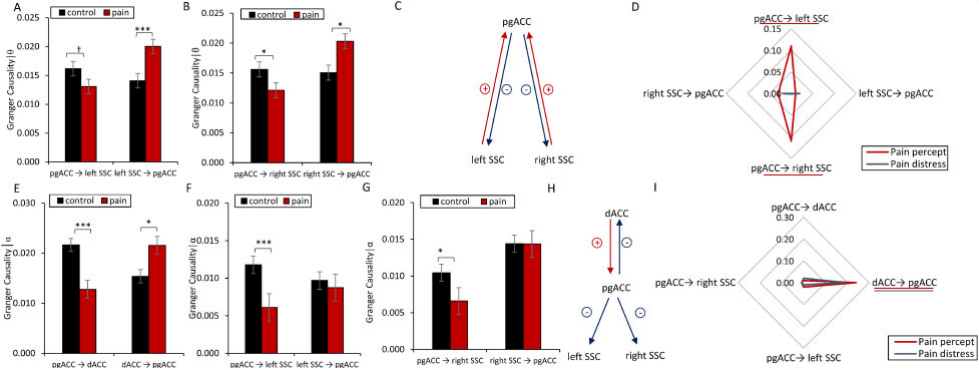
**Effective connectivity in pain**. (A–C) For the θ frequency band, a decrease in effective connectivity from the pregenual anterior cingulate cortex (pgACC) to the left and right the left and right somatosensory cortex (SCC) was identified, while an increase in effective connectivity from the left and right the left and right SCC to the pgACC was found for chronic pain patients in comparison to controls subjects. (D) A correlation analysis between pain intensity perception the left and right SCC to pgACC demonstrates that more pain is related to less effective connectivity in chronic pain patients. (E–H) For the α frequency band, a decrease in effective connectivity from the pgACC to the left and right SSC as well as the dorsal anterior cingulate cortex (dACC) was found for chronic pain patients. In contrast, no difference in effective connectivity from the left and right SCC to the dACC to the pgACC was noted for chronic pain patients in comparison to controls subjects. (I) A positive correlation was identified between pain perception with effective connectivity from dACC to pgACC, indicating the higher pain intensities and distress correlate with increased effective connectivity from dACC to pgACC in chronic pain patients. No effect was obtained for the effective connectivity from pgACC to SCC (left and right) or from pgACC to dACC. (†*P *<* *0.10, *P <* *0.05, **P <* *0.01, ***P < 0.001). For the radar plots, effective connectivity areas underlined indicate a significant effect (blue for pain distress, red for pain intensity).

For the θ band, an overall effect for effective connectivity as measured with Granger causality between the pgACC and the SSC (*F *=* *4.09, *P* < 0.004); Fig. 3a–c) was overall identified. A post-hoc univariate ANOVA revealed that there is less information sent from the pgACC to the left (*M* = 0.013, *SD* = 0.008; *F *=* *4.87, *P* = 0.041) and right SSC (*M* = 0.012, *SD* = 0.008; *F *=* *4.12, *P* = 0.045) for pain patients in comparison to the control subjects (left *M* = 0.016, *SD* = 0.008; right: *M* = 0.016, *SD* = 0.009). Increased information is transferred from both the left (*M* = 0.02 *SD* = 0.009; *F *=* *11.36, *P* < 0.001) and right SSC (*M* = 0.020, *SD* = 0.011; *F *=* *6.56, *P* = 0.012) to the pgACC for patients in comparison to control subjects (left *M* = 0.014, *SD* = 0.008; right: *M* = 0.015, *SD* = 0.009).

In patients with chronic pain both the information that is sent from pgACC to the left (*R*^2^ = 0.11, *P* = 0.042) and right SCC (*R*^2^ = 0.11, *P* = 0.041) negatively correlates with pain perception, as measured by the NRS. These effects, however, did not remain after correction for multiple comparison using a Bonferroni correction, No effect was obtained for the information that is sent from the left SSC (*R*^2^ = 0.01, *P* = 0.52) and right SCC (*R*^2^ = 0.03, *P* = 0.11) to pgACC. For pain distress, no significant correlations were obtained for pgACC to the left (*R*^2^ = 0.0001, *P* = 0.99) and right SCC (*R*^2^ = 0.001, *P* = 0.91) or from left SSC (*R*^2^ = 0.02, *P* = 0.88) and right SCC (*R*^2^ = 0.02, *P* = 0.39) to pgACC.

For the α band, an overall effect for the effective connectivity as measured with Granger causality was identified between the pgACC, dACC and the SSC (*F *=* *4.70, *P* < 0.001). A post-hoc univariate analysis indicated a significant increase (*F *=* *5.81, *P* = 0.018) in information flow from the dACC to the pgACC for pain patients (*M* = 0.021, *SD* = 0.011) in comparison to control subjects (*M* = 0.015, *SD* = 0.014). From the pgACC to the dACC, we found a significant decrease (*F *=* *22.48, *P* < 0.001) in information flow for pain patients (*M* = 0.013, *SD* = 0.008) in comparison to control subjects (*M* = 0.022, *SD* = 0.010). Furthermore, the analysis yielded a significant decrease for information flow from the pgACC to the left and right SCC for pain patients (left: *M* = 0.006, *SD* = 0.0008; *F *=* *11.94, *P* = 0.001; right: *M* = 0.007, *SD* = 0.007; *F *=* *6.29, *P* = 0.014) in comparison to control subjects (left: *M* = 0.012, *SD* = 0.008; right: *M* = 0.010, *SD* = 0.009). Univariate analyses revealed no effect for effective connectivity between the left SSC (*F* = 0.14, *P* = 0.71); and right SCC (*F* = 0.037, *P* = 0.85) to the pgACC. Also, for the left SSC (*F* = 0.10, *P* = 0.76); and right SCC (*F* = 0.12, *P* = 0.74) to dACC no effect was obtained. No effect was obtained for the dACC to the left SSC (*F* = 0.06, *P* = 0.82); and right SCC (*F* = 0.05, *P* = 0.83).

A correlation analysis revealed only a significant effect between pain intensity perception using the NRS and information flow from the dACC to the pgACC (*R*^2^ = 0.24, *P* < 0.001), indicating that the more pain was perceived by patients, the more information flowed from the dACC to the pgACC. No correlation was obtained for the pain percept and information flow from the pgACC to the dACC (*R*^2^ = 0.01, *P* = 0.90), from the pgACC to the left SSC (*R*^2^ = 0.02, *P* = 0.58) or from the pgACC to the right SSC (*R*^2^ = 0.01, *P* = 0.52), respectively. For pain distress, only a significant correlation was obtained between pain distress and information flow from the dACC to the pgACC (*R*^2^ = 0.19, *P* = 0.002). No correlation was obtained for the pain distress and information flow from the pgACC to the dACC (*R*^2^ = 0.02, *P* = 0.39), from the pgACC to the left SSC (*R*^2^ = 0.01, *P* = 0.50) or from the pgACC to the right SSC (*R*^2^ = 0.01, *P* = 0.53), respectively. The effect remained after correction for multiple comparison using a Bonferroni correction.

### Phase amplitude coupling

**Figure 4:**
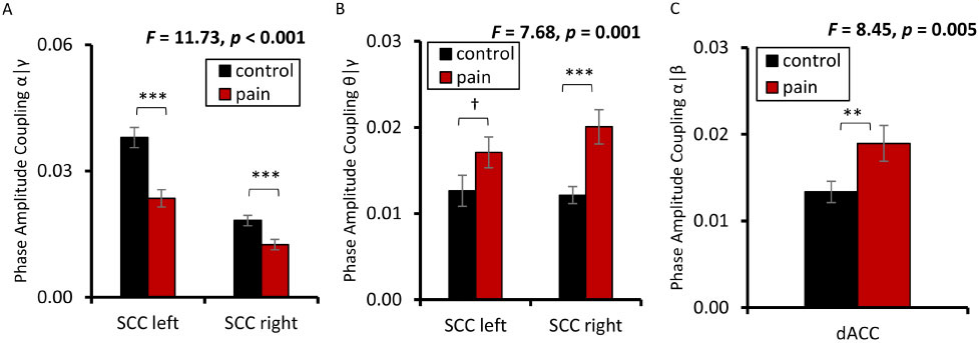
**Cross-frequency coupling in pain**. A significant decrease in α-γ coupling (a) and a significant increase in θ-γ coupling (b) was obtained for the left and right SCC for patients with chronic pain in comparison to control subjects. For the dACC, an increase in α-β coupling (c) was found for patients with chronic pain in comparison to control subjects. (†*P *<* *0.10, *P <* *0.05, **P <* *0.01, ***P < 0.001).

For θ-γ coupling, a significant difference was obtained for the left and right SSC (*F *=* *7.68, *P* < 0.001) between pain patients and control subjects. A post-hoc univariate analysis, revealed for right SSC (*M* = 0.020, *SD* = 0.012; *F *=* *14.64, *P* < 0.001) increased θ-γ coupling for pain patients in comparison to control subjects (*M* = 0.012, *SD* = 0.008). For the left SCC (*M* = 0.017, *SD* = 0.008; *F *=* *2.91, *P* = 0.091), there was only a marginally significant increase in θ-γ coupling for the pain patients in comparison to control subjects (*M* = 0.012, *SD* = 0.009 (Fig. 4a–c)).

No correlation was obtained between the pain intensity percept and θ-γ coupling for the left SSC (*R*^2^ = 0.03, *P* = 0.25) and right SSC (*R*^2^ = 0.002, *P* = 0.79). For pain distress, no correlation was obtained with θ-γ coupling for the left SSC (*R*^2^ = 0.01, *P* = 0.87) and right SSC (*R*^2^ = 0.001, *P* = 0.90).

For α-β coupling, a significant effect SSC (*F *=* *10.22, *P* < 0.001) was obtained of the dACC when comparing pain patients (*M* = 0.018, *SD* = 0.009) in comparison to control subjects (*M* = 0.013, *SD* = 0.008). No correlation was obtained between the pain percept and θ-γ coupling for (*R*^2^ = 0.001, *P* = 0.95) or between pain distress and θ-γ coupling (*R*^2^ = 0.001, *P* = 0.80) for the dACC.

## Discussion

This study provides converging evidence that chronic pain is an imbalance between brain areas involved in ascending and descending pain pathways. This imbalance is controlled by different brain oscillations that all contribute in routing of information flow between brain areas involved in pain input and pain suppression (Ploner *et al.*, 2017).

This study confirms that chronic pain is associated with increased θ and γ oscillations in the SSC, the main hub of the lateral ‘painfulness’ pathway, in agreement with the thalamocortical dysrhythmia model (Llinas *et al.*, 1999; Vanneste *et al.*, 2018), and specifies γ band activity in the somatosensory cortex correlates with intensity of chronic pain, similar to what is noted in acute experimental pain (Babiloni *et al.*, 2002).

The dACC, as main hub of the medial ‘suffering’ pathway, accelerates from θ to α and β frequencies, also suggesting an increase in pain processing.

The descending pain inhibitory system with the pgACC as main hub, which normally oscillates at rest in θ (Vanneste *et al.*, 2019). The results of this study demonstrate that the descending pain inhibitory systemaccelerate from θ to α suggesting that pain suppression is paradoxically increased, possibly as an attempt, albeit insufficient, to compensate for the increase in pain input. This strongly suggests that indeed a balance better explains pain than only pain input.

The imbalance can improve either by reducing pain input or increasing pain suppression. Spinal cord stimulation seems to rely on increased pgACC activity (Moens *et al.*, 2012), and by increasing pain suppression rebalancing pain suppression with pain input (De Ridder and Vanneste, 2016, 2017). This not only seems to be the main mechanism of action of spinal cord stimulation, but of motor cortex stimulation(Lima and Fregni, 2008). But also opioids (Fields, 2004) and SNRIs such as duloxetine(Lunn *et al.*, 2009; Kremer *et al.*, 2018) exert their pain suppressing effects by modulation of the descending pain inhibitory system. Furthermore, if the descending pain inhibitory system is deficient, as in fibromyalgia, people perceive generalized pain(Jensen *et al.*, 2013).

A balance, by definition, requires communication between pain input and suppression between the dACC, SSC, and pgACC. Thus, the lack of communication, as demonstrated by decreased functional connectivity between pain input and pain suppression, results in aloss of balance between interacting areas. Indeed, the results of this study indicate functional connectivity changes between dACC, SSC, and pgACC for both the θ and α band. A closer look at the data by analyzing effective connectivity indicates that for the θ band, less information is sent from the pgACC to the left and right SSC. This finding is associated with increased pain and fits with the idea that the imbalance is due to reduced pain suppression. For α, increased information is sent from the dACC to the pgACC, which correlates with the intensity of the pain percept. This latter finding could suggest that the dACC is inhibiting activity in the pgACC, explaining the reduced information being sent from the pgACC to the dACC, and the left and right SSC. Conceptually, the brain attaches a paradoxical behavioural significance to the pain percept, by which it remains conscious. Indeed fluctuations of the salience network results in conscious perception of somatosensory stimuli at threshold (Boly *et al.*, 2007). Furthermore, it has been shown that all psychosurgical interventions ultimately result in modulation of the pgACC (Schoene-Bake *et al.*, 2010). Thus a dACC cingulotomy will reduce the dACC part of the ratio, possibly normalizing the imbalance by releasing the pgACC from inhibitory activity (De Ridder *et al.*, 2016). And cingulotomies have been successfully used to treat chronic pain (Wilkinson *et al.*, 1999; Cetas *et al.*, 2008; Yen *et al.*, 2009).

The findings of this study also identify increased θ-γ coupling in the SSC, which is consistent with the thalamocortical dysrhythmia model of pain that proposes that α-γ nesting is a physiological mechanism transmitting sensory information and that slowed down alpha into the θ range reflects pathological θ-γ coupling, associated with tinnitus and pain (Llinas *et al.*, 1999; De Ridder *et al.*, 2015).

The balance disorder also suggests that there are multiple ways the brain can generate pain, either related to too much pain input, or because of deficient pain suppression, or a combination of the two. This could benefit a new brain-based classification of different types of pain, rather than the traditional etiology-based categorization of pain and may help to develop a mechanism-based approach for selecting therapy. Indeed, fibromyalgia pain has been attributed to a deficiency in pain suppression (Jensen *et al.*, 2013) and a balance problem between pain input and pain suppression (De Ridder and Vanneste, 2017), and this imbalance can be normalized by occipital nerve field stimulation (De Ridder and Vanneste, 2017). It is of interest that this imbalance mechanism might be more universal in view of the pathophysiological analogy between pain, tinnitus, Parkinson’s disease and major depression (Llinas *et al.*, 1999; Vanneste *et al.*, 2018) as well as reward-deficiency disorders, such as obesity, addiction, hyperactivity and personality disorders (Blum *et al.*, 2014). Indeed, the described mechanism involves the same brain areas involved in reward-deficiency disorders, resulting from allostasis, i.e. reference resetting (De Ridder *et al.*, 2016).

Furthermore, the fact that pain can be detected by machine learning with 89.3% accuracy, and if this concept is generalizable, will permit to develop objective measures for other subjective states such as tinnitus, depression, anxiety by measuring ratios of current density in the hubs that drive the ascending and descending pathways, rather than measuring electrical activity of only input areas of sensory pathways as was routinely done in for example tinnitus (van der Loo *et al.*, 2009), pain (Babiloni *et al.*, 2002) and depression (Jaworska *et al.*, 2012). The imbalance theory also permits to more rationally prescribe medications by clinicians. For example, combining medication that activate pain inhibition, with medications that inhibit pain input will be more effective in rebalancing pain or tinnitus or depression states than medication that only addresses one of the pain pathways. Thus, the magic bullet concept of Nobel laureate Paul Ehrlich (Winau *et al.*, 2004) does not hold in this concept, except if one medication would simultaneously modulate all three pathways. Ehrlich envisioned that just like a bullet fired from a gun aiming to hit a specific target, there must be a pharmacological way to specifically target a symptom or disease, in his case invading microbes. A more rational pain treatment however is to combine a drug that activates the descending pain inhibitory pathway, with another one that suppresses the medial pathway and a third that suppresses the lateral pathway. This cocktail approach is similar to what is very successfully used in for example AIDS pharmacotherapy, keeping the disease under control in 85–90% of patients (Lu *et al.*, 2018).

Although chronic pain is one of the most important medical problems facing society, there has been limited progress in finding an objective measure for this fundamentally subjective state. A conceptually new way of defining pain electrophysiologically may aid in developing better subtyping, better diagnostic measures and better treatments for chronic pain.

In conclusion, this paper presents a fundamentally new concept of pain as an imbalance disorder in the brain, which may have large implications, not only as a basis for finding an objective measure for a subjective pain state, but also for developing better pain medication, novel neurostimulation designs, and subtyping pain. Furthermore, in view of the analogy of the underlying pathophysiology of pain, tinnitus, depression, Parkinson’s disease (Vanneste *et al.*, 2018), and slow wave epilepsy (Llinas *et al.*, 1999), there is no reason to believe this concept could not be extended to other subjective states.

## Funding

No funding.

## Competing interests

The authors of no conflict of interests related to this research.

## Abbreviations

ADHD =: Attention Deficit Hyperactivity Disorder
dACC =: dorsal anterior cingulate cortex
EEG =: electroencephalogram
FRP =: false positive rate
MAE =: mean absolute error
MRI =: magnetic resonance imaging
NRS =: numeric rating scale
pgACC =: pregenual anterior cingulate cortex
PVAQ =: pain vigilance and awareness questionnaire
RMSE =: root mean squared error
ROC =: relative operating characteristic curve
sLORETA =: standardized low-resolution brain electromagnetic tomography
SSC =: somatosensory cortex
SVM =: support vector machine
TPR =: true positive rate

## References

[fcab014-B1] Babiloni C, Babiloni F, Carducci F, Cincotti F, Rosciarelli F, Arendt-Nielsen L, et al Human brain oscillatory activity phase-locked to painful electrical stimulations: a multi-channel EEG study. Hum Brain Mapp 2002; 15: 112–23. 11835602 10.1002/hbm.10013 PMC6872083

[fcab014-B2] Blum K, Oscar-Berman M, Demetrovics Z, Barh D, Gold MS. Genetic Addiction Risk Score (GARS): molecular neurogenetic evidence for predisposition to Reward Deficiency Syndrome (RDS). Mol Neurobiol 2014; 50: 765–96. 24878765 10.1007/s12035-014-8726-5 PMC4225054

[fcab014-B3] Boly M, Balteau E, Schnakers C, Degueldre C, Moonen G, Luxen A, et al Baseline brain activity fluctuations predict somatosensory perception in humans. Proc Natl Acad Sci U S A 2007; 104: 12187–92. 17616583 10.1073/pnas.0611404104 PMC1924544

[fcab014-B4] Bushnell MC, Čeko M, Low LA. Cognitive and emotional control of pain and its disruption in chronic pain. Nat Rev Neurosci 2013; 14: 502–11. 23719569 10.1038/nrn3516 PMC4465351

[fcab014-B5] Canolty RT, Edwards E, Dalal SS, Soltani M, Nagarajan SS, Kirsch HE, et al High gamma power is phase-locked to theta oscillations in human neocortex. Science 2006; 313: 1626–8. 16973878 10.1126/science.1128115 PMC2628289

[fcab014-B6] Cauda F, Sacco K, D'Agata F, Duca S, Cocito D, Geminiani G, et al Low-frequency BOLD fluctuations demonstrate altered thalamocortical connectivity in diabetic neuropathic pain. BMC Neurosci 2009; 10: 138. 19941658 10.1186/1471-2202-10-138 PMC2789078

[fcab014-B7] Cetas JS, Saedi T, Burchiel KJ. Destructive procedures for the treatment of nonmalignant pain: a structured literature review. JNS 2008; 109: 389–404. 10.3171/JNS/2008/109/9/0389 18759567

[fcab014-B8] Coullaut-Valera R, Arbaiza I, Bajo R, Arrue R, Lopez ME, Coullaut-Valera J, et al Drug polyconsumption is associated with increased synchronization of brain electrical-activity at rest and in a counting task. Int J Neural Syst 2014; 24: 1450005. 24344693 10.1142/S0129065714500051

[fcab014-B9] De Ridder D, Manning P, Leong SL, Ross S, Vanneste S. Allostasis in health and food addiction. Sci Rep 2016; 6: 37126. 27876789 10.1038/srep37126 PMC5120365

[fcab014-B10] De Ridder D, van der Loo E, Vanneste S, Gais S, Plazier M, Kovacs S, et al Theta-gamma dysrhythmia and auditory phantom perception. JNS 2011; 114: 912–21. 10.3171/2010.11.JNS10335 21235308

[fcab014-B11] De Ridder D, Vanneste S. Burst and tonic spinal cord stimulation: different and common brain mechanisms. Neuromodulation 2016; 19: 47–59. 26586145 10.1111/ner.12368

[fcab014-B12] De Ridder D, Vanneste S. Occipital nerve field transcranial direct current stimulation normalizes imbalance between pain detecting and pain inhibitory pathways in fibromyalgia. Neurotherapeutics 2017; 14: 484–501. 28004273 10.1007/s13311-016-0493-8 PMC5398977

[fcab014-B13] De Ridder D, Vanneste S, Gillett G, Manning P, Glue P, Langguth B. Psychosurgery reduces uncertainty and increases free will? A review. Neuromodulation 2016; 19: 239–48. 26899938 10.1111/ner.12405

[fcab014-B14] De Ridder D, Vanneste S, Langguth B, Llinas R. Thalamocortical dysrhythmia: a theoretical update in tinnitus. Front Neurol 2015; 6: 124 26106362 10.3389/fneur.2015.00124 PMC4460809

[fcab014-B15] de Vries M, Wilder-Smith OH, Jongsma ML, van den Broeke EN, Arns M, van Goor H, et al Altered resting state EEG in chronic pancreatitis patients: toward a marker for chronic pain. J Pain Res 2013; 6: 815–24. 24379694 10.2147/JPR.S50919 PMC3843642

[fcab014-B16] Eippert F, Bingel U, Schoell ED, Yacubian J, Klinger R, Lorenz J, et al Activation of the opioidergic descending pain control system underlies placebo analgesia. Neuron 2009; 63: 533–43. 19709634 10.1016/j.neuron.2009.07.014

[fcab014-B17] Fields H. State-dependent opioid control of pain. Nat Rev Neurosci 2004; 5: 565–75. 15208698 10.1038/nrn1431

[fcab014-B18] Flor H, Elbert T, Knecht S, Wienbruch C, Pantev C, Birbaumer N, et al Phantom-limb pain as a perceptual correlate of cortical reorganization following arm amputation. Nature 1995; 375: 482–4. 7777055 10.1038/375482a0

[fcab014-B19] Fuchs M, Kastner J, Wagner M, Hawes S, Ebersole JS. A standardized boundary element method volume conductor model. Clin Neurophysiol 2002; 113: 702–12. 11976050 10.1016/s1388-2457(02)00030-5

[fcab014-B20] Gaskin DJ, Richard P. The economic costs of pain in the United States. J Pain 2012; 13: 715–24. 22607834 10.1016/j.jpain.2012.03.009

[fcab014-B21] Geweke J. Measurement of lineair dependence and feedback between multiple time series. J Am Stat Assoc 1982; 77: 304–13.

[fcab014-B22] Granger CWJ. Investigating causal relations by econometrics models and crosss-spectral methods. Econometrica 1969; 37: 424.

[fcab014-B23] Jaworska N, Blier P, Fusee W, Knott V. Alpha power, alpha asymmetry and anterior cingulate cortex activity in depressed males and females. J Psychiatr Res 2012; 46: 1483–91. 22939462 10.1016/j.jpsychires.2012.08.003 PMC3463760

[fcab014-B24] Jensen KB, Srinivasan P, Spaeth R, Tan Y, Kosek E, Petzke F, et al Overlapping structural and functional brain changes in patients with long-term exposure to fibromyalgia pain. Arthritis Rheum 2013; 65: 3293–303. 23982850 10.1002/art.38170 PMC3984030

[fcab014-B25] Jensen MP, Day MA, Miro J. Neuromodulatory treatments for chronic pain: efficacy and mechanisms. Nat Rev Neurol 2014; 10: 167–78. 24535464 10.1038/nrneurol.2014.12 PMC5652321

[fcab014-B26] Jensen MP, Sherlin LH, Gertz KJ, Braden AL, Kupper AE, Gianas A, et al Brain EEG activity correlates of chronic pain in persons with spinal cord injury: clinical implications. Spinal Cord 2013; 51: 55–8. 22801188 10.1038/sc.2012.84

[fcab014-B27] Jurcak V, Tsuzuki D, Dan I. 10/20, 10/10, and 10/5 systems revisited: their validity as relative head-surface-based positioning systems. Neuroimage 2007; 34: 1600–11. 17207640 10.1016/j.neuroimage.2006.09.024

[fcab014-B28] Kim JW, Kim SY, Choi JW, Kim KM, Nam SH, Min KJ, et al Differences in resting-state quantitative electroencephalography patterns in attention deficit/hyperactivity disorder with or without comorbid symptoms. Clin Psychopharmacol Neurosci 2017; 15: 138–45. 28449561 10.9758/cpn.2017.15.2.138 PMC5426496

[fcab014-B29] Kim JW, Lee J, Kim HJ, Lee YS, Min KJ. Relationship between theta-phase gamma-amplitude coupling and attention-deficit/hyperactivity behavior in children. Neurosci Lett 2015; 590: 12–7. 25637702 10.1016/j.neulet.2015.01.068

[fcab014-B30] Kong J, Loggia ML, Zyloney C, Tu P, Laviolette P, Gollub RL. Exploring the brain in pain: activations, deactivations and their relation. Pain 2010; 148: 257–67. 20005043 10.1016/j.pain.2009.11.008 PMC2815185

[fcab014-B31] Kremer M, Yalcin I, Goumon Y, Wurtz X, Nexon L, Daniel D, et al A dual noradrenergic mechanism for the relief of neuropathic allodynia by the antidepressant drugs duloxetine and amitriptyline. J Neurosci 2018; 38: 9934–54. 30249798 10.1523/JNEUROSCI.1004-18.2018 PMC6596240

[fcab014-B32] Lima MC, Fregni F. Motor cortex stimulation for chronic pain: systematic review and meta-analysis of the literature. Neurology 2008; 70: 2329–37. 18541887 10.1212/01.wnl.0000314649.38527.93

[fcab014-B33] Lisman JE, Jensen O. The theta-gamma neural code. Neuron 2013; 77: 1002–16. 23522038 10.1016/j.neuron.2013.03.007 PMC3648857

[fcab014-B34] Llinas R, Urbano FJ, Leznik E, Ramirez RR, van Marle HJ. Rhythmic and dysrhythmic thalamocortical dynamics: GABA systems and the edge effect. Trends Neurosci 2005; 28: 325–33. 15927689 10.1016/j.tins.2005.04.006

[fcab014-B35] Llinas RR, Ribary U, Jeanmonod D, Kronberg E, Mitra PP. Thalamocortical dysrhythmia: a neurological and neuropsychiatric syndrome characterized by magnetoencephalography. Proc Natl Acad Sci U S A 1999; 96: 15222–7. 10611366 10.1073/pnas.96.26.15222 PMC24801

[fcab014-B36] Logan JM, Sanders AL, Snyder AZ, Morris JC, Buckner RL. Under-recruitment and nonselective recruitment: dissociable neural mechanisms associated with aging. Neuron 2002; 33: 827–40. 11879658 10.1016/s0896-6273(02)00612-8

[fcab014-B37] Lu DY, Wu HY, Yarla NS, Xu B, Ding J, Lu TR. HAART in HIV/AIDS treatments: future trends. Infect Disord Drug Targets 2018; 18: 15–22. 28474549 10.2174/1871526517666170505122800

[fcab014-B38] Lunn MP, Hughes RA, Wiffen PJ. Duloxetine for treating painful neuropathy or chronic pain. Cochrane Database Syst Rev 2009; CD007115. 19821395 10.1002/14651858.CD007115.pub2

[fcab014-B39] Moazami-Goudarzi M, Michels L, Weisz N, Jeanmonod D. Temporo-insular enhancement of EEG low and high frequencies in patients with chronic tinnitus. QEEG study of chronic tinnitus patients. BMC Neurosci 2010; 11: 40. 20334674 10.1186/1471-2202-11-40 PMC2858736

[fcab014-B40] Moens M, Sunaert S, Marien P, Brouns R, De Smedt A, Droogmans S, et al Spinal cord stimulation modulates cerebral function: an fMRI study. Neuroradiology 2012; 54: 1399–407. 22941431 10.1007/s00234-012-1087-8

[fcab014-B41] Mumtaz W, Vuong PL, Xia L, Malik AS, Rashid RBA. An EEG-based machine learning method to screen alcohol use disorder. Cogn Neurodyn 2017; 11: 161–71. 28348647 10.1007/s11571-016-9416-y PMC5350086

[fcab014-B42] Pascual-Marqui R, (2007a). Discrete, 3D distributed, linear imaging methods of electric neuronal activity. Part 1: exact, zero error localization.https://arxiv.org/abs/0710.3341 (23 February 2021, date last accessed).

[fcab014-B43] Pascual-Marqui R, (2007b). Instantaneous and lagged measurements of linear and nonlinear dependence between groups of multivariate time series: frequency decomposition.https://arxiv.org/abs/0711.1455 (23 February 2021, date last accessed).

[fcab014-B44] Pascual-Marqui RD. Standardized low-resolution brain electromagnetic tomography (sLORETA): technical details. Methods Find Exp Clin Pharmacol 2002; 24 Suppl D: 5–12. 12575463

[fcab014-B45] Ploner M, Sorg C, Gross J. Brain rhythms of pain. Trends Cogn Sci 2017; 21: 100–10. 28025007 10.1016/j.tics.2016.12.001 PMC5374269

[fcab014-B46] Price DD. Psychological and neural mechanisms of the affective dimension of pain. Science 2000; 288: 1769–72. 10846154 10.1126/science.288.5472.1769

[fcab014-B47] Rainville P, Duncan GH, Price DD, Carrier B, Bushnell MC. Pain affect encoded in human anterior cingulate but not somatosensory cortex. Science 1997; 277: 968–71. 9252330 10.1126/science.277.5328.968

[fcab014-B48] Roelofs J, Peters ML, McCracken L, Vlaeyen JW. The pain vigilance and awareness questionnaire (PVAQ): further psychometric evaluation in fibromyalgia and other chronic pain syndromes. Pain 2003; 101: 299–306. 12583873 10.1016/S0304-3959(02)00338-X

[fcab014-B49] Sametsky EA, Turner JG, Larsen D, Ling L, Caspary DM. Enhanced GABAA-mediated tonic inhibition in auditory thalamus of rats with behavioral evidence of tinnitus. J Neurosci 2015; 35: 9369–80. 26109660 10.1523/JNEUROSCI.5054-14.2015 PMC4478253

[fcab014-B50] Sarnthein J, Morel A, von Stein A, Jeanmonod D. Thalamocortical theta coherence in neurological patients at rest and during a working memory task. Int J Psychophysiol 2005; 57: 87–96. 15982767 10.1016/j.ijpsycho.2005.03.015

[fcab014-B51] Sarnthein J, Stern J, Aufenberg C, Rousson V, Jeanmonod D. Increased EEG power and slowed dominant frequency in patients with neurogenic pain. Brain 2006; 129: 55–64. 16183660 10.1093/brain/awh631

[fcab014-B52] Schmidt S, Naranjo JR, Brenneisen C, Gundlach J, Schultz C, Kaube H, et al Pain ratings, psychological functioning and quantitative EEG in a controlled study of chronic back pain patients. PLoS One 2012; 7: e31138. 22431961 10.1371/journal.pone.0031138 PMC3303776

[fcab014-B53] Schoene-Bake JC, Parpaley Y, Weber B, Panksepp J, Hurwitz TA, Coenen VA. Tractographic analysis of historical lesion surgery for depression. Neuropsychopharmacology 2010; 35: 2553–63. 20736994 10.1038/npp.2010.132 PMC3055575

[fcab014-B54] Schulman JJ, Cancro R, Lowe S, Lu F, Walton KD, Llinas RR. Imaging of thalamocortical dysrhythmia in neuropsychiatry. Front Hum Neurosci 2011; 5: 69 21863138 10.3389/fnhum.2011.00069 PMC3149146

[fcab014-B55] Siepmann M, Kirch W. Effects of caffeine on topographic quantitative EEG. Neuropsychobiology 2002; 45: 161–6. 11979068 10.1159/000054958

[fcab014-B56] Smith TC, Frank E.Introducing machine learning concepts with WEKA. In: Mathe E, Davis S, editors. Statiscal genomics: methods and protocols. New York, USA: Springer; 2016. p. 348–353. 10.1007/978-1-4939-3578-9_17 27008023

[fcab014-B57] Song JJ, Vanneste S, De Ridder D. Dysfunctional noise cancelling of the rostral anterior cingulate cortex in tinnitus patients. PLoS One 2015; 10: e0123538. 25875099 10.1371/journal.pone.0123538 PMC4395158

[fcab014-B58] Stern J, Jeanmonod D, Sarnthein J. Persistent EEG overactivation in the cortical pain matrix of neurogenic pain patients. Neuroimage 2006; 31: 721–31. 16527493 10.1016/j.neuroimage.2005.12.042

[fcab014-B59] Treede RD, Rief W, Barke A, Aziz Q, Bennett MI, Benoliel R, et al A classification of chronic pain for ICD-11. Pain 2015; 156: 1003–7. 25844555 10.1097/j.pain.0000000000000160 PMC4450869

[fcab014-B60] van der Loo E, Gais S, Congedo M, Vanneste S, Plazier M, Menovsky T, et al Tinnitus intensity dependent gamma oscillations of the contralateral auditory cortex. PLoS One 2009; 4: e7396.7391–7395. 19816597 10.1371/journal.pone.0007396 PMC2754613

[fcab014-B61] Vanneste S, Alsalman O, De Ridder D. Top-down and bottom-up regulated auditory phantom perception. J Neurosci 2019; 39: 364–78. 30389837 10.1523/JNEUROSCI.0966-18.2018 PMC6360282

[fcab014-B62] Vanneste S, Congedo M, De Ridder D. Pinpointing a highly specific pathological functional connection that turns phantom sound into distress. Cereb Cortex 2014; 24: 2268–82. 23632885 10.1093/cercor/bht068

[fcab014-B63] Vanneste S, Song JJ, De Ridder D. Thalamocortical dysrhythmia detected by machine learning. Nat Commun 2018; 9: 1103. 29549239 10.1038/s41467-018-02820-0 PMC5856824

[fcab014-B64] Volkow ND, Logan J, Fowler JS, Wang GJ, Gur RC, Wong C, et al Association between age-related decline in brain dopamine activity and impairment in frontal and cingulate metabolism. Am J Psychiatry 2000; 157: 75–80. 10618016 10.1176/ajp.157.1.75

[fcab014-B65] Walton KD, Dubois M, Llinas RR. Abnormal thalamocortical activity in patients with Complex Regional Pain Syndrome (CRPS) type I. Pain 2010; 150: 41–51. 20338687 10.1016/j.pain.2010.02.023

[fcab014-B66] Wilkinson HA, Davidson KM, Davidson RI. Bilateral anterior cingulotomy for chronic noncancer pain. Neurosurgery 1999; 45: 1129–34. discussion 1134–1126. 10549929 10.1097/00006123-199911000-00023

[fcab014-B67] Winau F, Westphal O, Winau R. Paul Ehrlich–in search of the magic bullet. Microbes Infect 2004; 6: 786–9. 15207826 10.1016/j.micinf.2004.04.003

[fcab014-B68] Yen CP, Kuan CY, Sheehan J, Kung SS, Wang CC, Liu CK, et al Impact of bilateral anterior cingulotomy on neurocognitive function in patients with intractable pain. J Clin Neurosci 2009; 16: 214–9. 19101146 10.1016/j.jocn.2008.04.008

[fcab014-B69] Yilmaz P, Diers M, Diener S, Rance M, Wessa M, Flor H. Brain correlates of stress-induced analgesia. Pain 2010; 151: 522–9. 20817354 10.1016/j.pain.2010.08.016

